# Total Iron Absorbed from Iron-Biofortified Potatoes Is Higher than that from Nonbiofortified Potatoes: A Randomized Trial Using Stable Iron Isotopes in Women from the Peruvian Highlands

**DOI:** 10.1016/j.tjnut.2023.04.010

**Published:** 2023-04-13

**Authors:** Gabriela Burgos, Reyna Liria, Christophe Zeder, Paul A. Kroon, Guy Hareau, Mary Penny, Jack Dainty, Olla Al-Jaibaji, Erick Boy, Richard Mithen, Richard F. Hurrell, Elisa Salas, Thomas zum Felde, Michael B. Zimmermann, Susan Fairweather-Tait

**Affiliations:** 1 Genetics, Genomics, and Crop Improvement Program, International Potato Center, Lima, Peru; 2 Instituto de Investigación Nutricional, Lima, Peru; 3 ETH Zürich, Laboratory of Human Nutrition, Institute of Food, Nutrition, and Health, Department of Health Sciences and Technology, Zurich, Switzerland; 4 Quadram Institute Bioscience, Norwich Research Park, Norwich, United Kingdom; 5 Norwich Medical School, University of East Anglia, Norwich, United Kingdom; 6 HarvestPlus /International Food Policy Research Institute, Washington, DC, United States; 7 Liggins Institute, Waipapa Taumata Rau - The University of Auckland, Auckland, New Zealand

**Keywords:** iron-biofortified potato, biofortified crop, iron absorption, bioavailability, women, stable isotopes, Latin America

## Abstract

**Background:**

Yellow-fleshed potatoes biofortified with iron have been developed through conventional breeding, but the bioavailability of iron is unknown.

**Objectives:**

Our objective was to measure iron absorption from an iron-biofortified yellow-fleshed potato clone in comparison with a nonbiofortified yellow-fleshed potato variety.

**Methods:**

We conducted a single-blinded, randomized, crossover, multiple-meal intervention study. Women (*n* = 28; mean ± SD plasma ferritin 21.3 ± 3.3 μg/L) consumed 10 meals (460 g) of both potatoes, each meal extrinsically labeled with either ^58^Fe sulfate (biofortified) or ^57^Fe sulfate (nonfortified), on consecutive days. Iron absorption was estimated from iron isotopic composition in erythrocytes 14 d after administration of the final meal.

**Results:**

Mean ± SD iron, phytic acid, and ascorbic acid concentrations in iron-biofortified and the nonfortified potato meals (mg/per 100 mg) were 0.63 ± 0.01 and 0.31 ± 0.01, 39.34 ± 3.04 and 3.10 ± 1.72, and 7.65 ± 0.34 and 3.74 ± 0.39, respectively (*P* < 0.01), whereas chlorogenic acid concentrations were 15.14 ± 1.72 and 22.52 ± 3.98, respectively (*P* < 0.05). Geometric mean (95% CI) fractional iron absorption from the iron-biofortified clone and the nonbiofortified variety were 12.1% (10.3%–14.2%) and 16.6% (14.0%–19.6%), respectively (*P* < 0.001). Total iron absorption from the iron-biofortified clone and the nonbiofortified variety were 0.35 mg (0.30–0.41 mg) and 0.24 mg (0.20–0.28 mg) per 460 g meal, respectively (*P* < 0.001).

**Conclusions:**

TIA from iron-biofortified potato meals was 45.8% higher than that from nonbiofortified potato meals, suggesting that iron biofortification of potatoes through conventional breeding is a promising approach to improve iron intake in iron-deficient women.

The study was registered at www.clinicaltrials.gov as Identifier number NCT05154500.

## Introduction

Iron deficiency anemia is a global problem affecting 2 billion people worldwide, particularly women and children [1]. In Peru, anemia is still pervasive with a prevalence of 39% for children aged 6 to 35 mo, and 19% for women between 15 and 49 y. Anemia is even more common in rural highland areas, averaging 49% for children and 20% for women [2], and this constitutes an undesirable burden to smallholding farmers and their families.

Biofortification is the process of increasing the density of micronutrients in food crops through conventional plant breeding, agronomic practices, or genetic modification [3]. Biofortification of staple crops is considered an attractive and sustainable agricultural strategy to deliver essential micronutrients to malnourished populations with limited access to other micronutrient interventions (such as supplementation or food fortification). Biofortified crops are particularly advantageous for improving the nutritional status of people in rural food systems in low- and middle-income countries, where diets of farming families are heavily dependent on their own produce or locally procured staple crops and where the prevalence of micronutrient (iron, zinc, and vitamin A) deficiencies is high [4].

Potatoes are the fourth most important crop in Latin America, where 57 million people suffer from iron deficiency [5]. In the Peruvian highlands, potatoes are the backbone of agriculture and diets and are considered a staple crop with a mean consumption in rural areas reaching 800 g and 200 g potato per day for women and children, respectively [6].

For >18 y, the International Potato Center (CIP) has been using conventional breeding methods to develop iron-biofortified potatoes to help reduce iron deficiency. Iron-biofortified potatoes contain around 50% more iron than the current varieties grown in target countries and can withstand major potato pests and diseases. They have a yield similar to most commercial potato varieties and satisfy the preferences of farmers and consumers [7].

The nutritional value of iron in potatoes is not widely recognized because when compared with legumes and cereals, the amounts are low. However, potatoes contain significant amounts of ascorbic acid, a promoter of iron absorption together with low levels of phytate, an inhibitor of iron absorption [8]. According to a recent absorption study in women, the iron absorption from a yellow-fleshed potato was higher (29%) than that from a purple-fleshed potato (13%), presumably because of inhibitory phenolics in the purple potatoes [9]. Nevertheless, iron absorption in both types of potatoes is substantially higher than that reported for other iron-biofortified crops such as pearl millet (7.5%) [10] and beans (3.4%–4.7%) [11].

The aim of this study was to assess iron bioavailability from a yellow-fleshed iron-biofortified potato compared with a yellow-fleshed commercial potato variety by measuring iron absorption from stable isotope incorporation into erythrocytes after consumption of multiple isotopically labeled test meals.

## Methods

### Study site and subjects

The study was conducted from August to December 2021 in Huancavelica (altitude, 3600 m), a city located in the central highlands of Peru.

**FIGURE 1:**
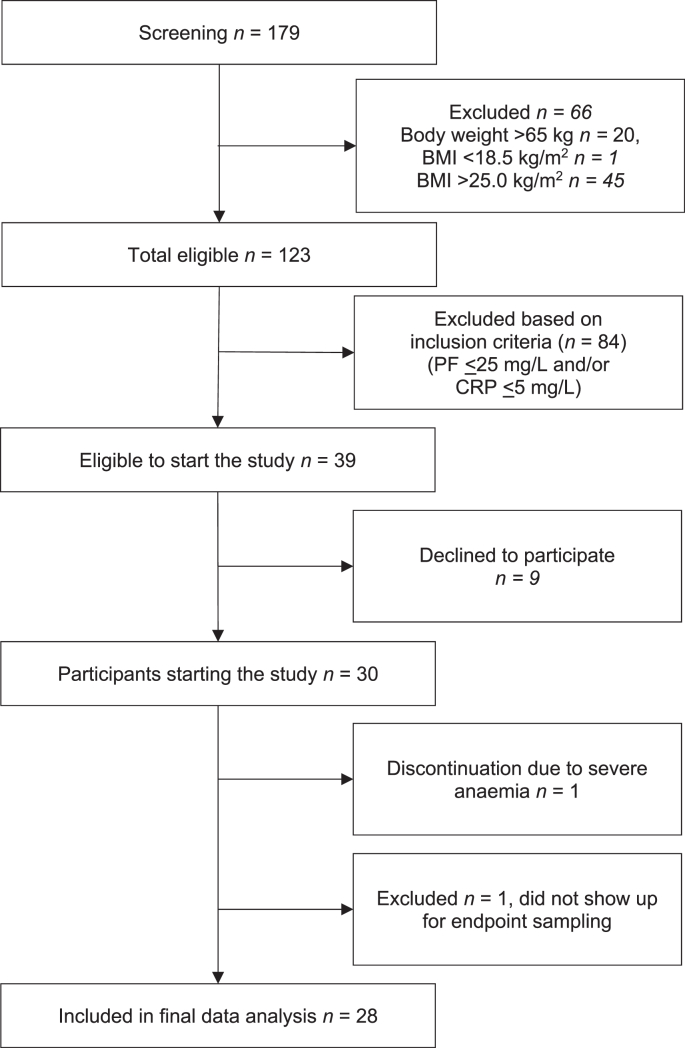
Study overview diagram.∗ BMI, body mass index; CRP, C-reactive protein; PF, plasma ferritin

Interested participants were initially screened by using the inclusion criteria of age, weight, and height. A total of 123 eligible women were invited for a full screening. Thirty-nine participants were eligible but 9 participants declined to participate in the study. Thirty participants started the study and 28 participants completed the study (Figure 1). The inclusion criteria were as follows: body weight (<65 kg), BMI (kg/m^2^; 18.5–25.0), age (18–25 y), plasma ferritin (PF) concentration ≤25 μg/L, C-reactive protein (CRP) concentration <5 mg/L, and hemoglobin (Hb) >80 g/L, adjusted for altitude [12], not pregnant (assessed by administering a urine pregnancy test) or lactating, no chronic diseases or medications that could influence iron metabolism, no current intake of mineral and vitamin supplements or willing to discontinue supplementation 2 wk before study start, no current participation in any clinical trial or during the past month before study start, no presence of fever (>37.5°C) on the first study day, and being able to attend all study visits for ∼4 wk (total 20 d) and, consume each day the prepared potatoes and stay for a further 3 h after finishing the meal.

### Study design

This study follows a single-blinded, randomized, crossover design in which each subject consumes a sequence of 2 types of meals (biofortified and nonbiofortified potatoes). Each type of potato is labeled with a different stable iron isotope (^58^Fe for the biofortified variety and ^57^Fe for the nonbiofortified variety). The isotopic labels are absorbed and incorporated into the erythrocytes. Fourteen days after the last administration, the amount of isotopic label (calculated from the isotopic composition) in the erythrocytes reaches a plateau, which remains stable in the following weeks or even months. The use of 2 different isotopic tracers allows the calculation of fractional iron absorption (FIA) and total iron absorption (TIA) of the 2 potato varieties in a single blood sample, taken 14 d after the last administration of the test meals (day 40 of the study).

**FIGURE 2:**
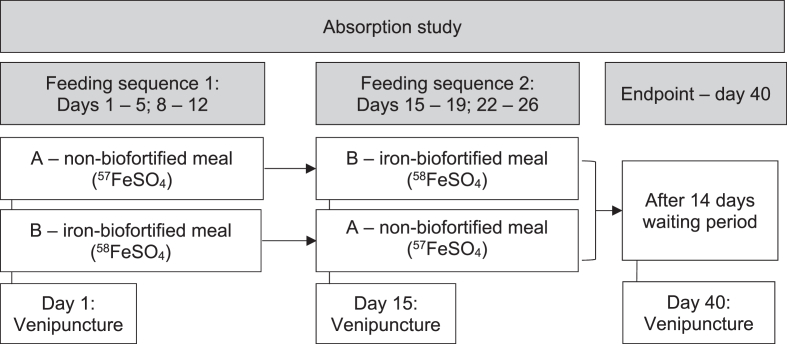
Study design for the potato study in Peru. Participants were randomly assigned to either the nonbiofortified or iron-biofortified test meals with iron isotopic administration (nonbiofortified test meal, ^57^Fe, or iron-biofortified test meal, ^58^Fe) for 10 d, after which they changed to the other test meal sequence.

A venous blood sample was collected on the first study day to determine the baseline iron isotopic composition as well as iron and inflammation status markers. Women began consuming the first randomly assigned potato meal labeled with 0.3 mg of the corresponding iron isotope: ^57^Fe or ^58^Fe for the nonbiofortified and biofortified potato, respectively (Figure 2), and after this, they continued with the second potato meal. Women were assigned to the nonbiofortified or biofortified potato meal using a simple random assignment. For this purpose, each participant was given a number according to the order of enrollment, and random numbers were generated by the trial supervisor using Excel (Microsoft 365 MSO (Version 2202). At the end of the intervention, all participants had consumed both types of potato meals, so each participant acted as her own control.

Participants were blinded so they did not know the order in which the test meals were given. The first assigned test meal was consumed as breakfast for 10 consecutive days but was interrupted by a weekend break. On day 15, all participants switched over to the second test meal type, which they consumed for another 10 d. Each test meal consisted of 460 g of mashed potato served in 2 bowls of 230 g each. The test meals were weighed before and after consumption to calculate the intrinsic iron intake. The stable isotope solution was given in 50 mL water, which was drunk after the first 230-g portion had been consumed. In addition, each participant received 200 to 400 mL of mineral water.

Meals were consumed under direct supervision by the study team after overnight fasting was confirmed. Participants were observed for 3 h after the test meals, and no food or drink was allowed other than mineral water (500 mL). Venous blood samples were collected by trained staff on days 15 and 40 to measure iron and markers of inflammation, and isotopic composition was measured in the days 1 and 40 samples. Subjects developing a fever or other symptoms of illness were discontinued from the study and advised to consult their medical doctor. The study was conducted under pandemic restrictions, respecting social distance, and all the measures adopted by the Peruvian government during the time of the study. The feeding trial had a duration of 20 wk (August to December 2021).

### Potato samples and meal preparation

The iron-biofortified potato clone CIP311623.75 and the nonbiofortified commercial potato variety Peruanita were grown in Paucartambo, Pasco, Peru. The potatoes were harvested in May 2021 and were transported to Lima where they were prepared in June.

Briefly, the potato tubers were washed under running water and boiled in 6 kg batches, completely covered in water, for 60 min after which the potatoes were peeled and mashed. The mash was stored at –20C° in plastic bags until the COVID restrictions allowed us to start the intervention. The potato meals were transported frozen from Lima to Huancavelica in 3 batches.

The night before the consumption of test meals, the frozen potato meals were taken out of the freezer, left at room temperature (2°C) overnight, and warmed in a microwave oven before being served to the participants.

### Stable isotope tracers

The stable isotope labels, ^57^FeSO_4_ and ^58^FeSO_4_, were prepared from ^57^Fe-enriched elemental iron (96.2% isotopic enrichment) and ^58^Fe-enriched elemental iron (95.3% isotopic enrichment; Chemgas, Boulogne, France) by dissolution in 2 M sulfuric acid and subsequent dilution with ultra-pure water to a concentration of 0.6 mg Fe/g solution. The vials were flushed with argon to avoid the oxidation of Fe(II) to Fe(III). Each vial contained 3 mg of labeled iron, sufficient to label 10 meals of each type for each participant. The vials were stored at the study site at 2 to 8°C until use.

### Test meal analyses

Meal analyses were performed in freeze-dried, ground potato meal samples. The analysis (except for ascorbic acid) was performed on test meals prepared using a subset of the tubers used for the preparation of the test meals, 2 wk before the preparation of all the test meals. To test for variation during processing and storage, ascorbic acid was analyzed in cooked and mashed tubers immediately after preparation, and also in cooked and mashed tuber samples after storing at −20°C for 5 mo and thawing at room temperature overnight.

Grinding of samples was done in a stainless-steel mill only used for potato samples to avoid sample contamination. Iron concentration was measured using a method based on Mwesigye et al. [13] that briefly consist of digesting the sample by refluxing using HNO_3_ in a hot plate for 2 h, and analysis by ICP–MS (Thermo-Fisher Scientific iCAP-Q; Thermo-Fisher Scientific, Bremen, Germany). Phytic acid (PA) concentration was measured using a modification of the Makower method [14], as described previously by Paganini et al. [15]. Individual polyphenol concentration was determined following a method based on Deußer et al. [16], which includes methanolic extraction and analysis using ultraperformance liquid chromatography with mass spectrometry. The mass spectrometry-based approach applied utilized a triple quadrupole and targeted multiple reaction monitoring. Total polyphenols are the sum of individual phenolics. Ascorbic acid was analyzed by UV/Visible spectroscopy (UV 160A; Shimadzu Corp), as described by Burgos et al. [17].

### Blood analysis

For screening, whole blood samples were analyzed for Hb using a 5-differential flow cytometer (Nihon Kohden). PF and CRP were analyzed using electrochemiluminescence (Cobas E 411; Roche Diagnostics), and turbidimetry (Vitros 4600; Ortho Clinical Diagnostics), respectively.

Whole blood and plasma samples were collected from participants and shipped from Lima to the Human Nutrition Laboratory of ETH Zurich on dry ice and stored at −20°C until analysis. PF and CRP concentrations were measured in the samples using an automated immunoassay analyzer (Immulite 1000, Siemens Healthineers, Germany). Accuracy was checked by measuring control samples (CRP 3 levels, PF 2 levels) from the same manufacturer. Whole blood samples were mineralized with concentrated nitric acid in a microwave autoclave (TurboWave, MLS, Germany). The iron present in the mineralized samples was isolated by anion exchange chromatography and subsequently purified by precipitation as ferric hydroxide [18]. The isotopic composition of the isolated iron was measured by multicollector inductively coupled plasma mass spectrometry (Neptune, Thermo-Fisher Scientific, Bremen Germany) [18].

### Calculation of FIA

FIA was calculated according to the principle of isotopic dilution, based on the shifts of isotopic ratios between the baseline and endpoint, and the amount of circulating iron in the body [19]. Circulating iron was estimated from blood volume [20], and the mean Hb concentration on days 1, 15, and 40. An 80% incorporation rate of the isotopic labels into the erythrocytes was assumed [21].

### Sample size calculation

Assuming a standard deviation (SD) of 0.18 (SD of the log-transformed differences within pairs from a similar previous iron absorption study [19]), a type I error rate of 5% and 80% power, a difference in the total iron absorbed of 35% can be detected with a sample size of 20 participants. To allow for potential dropouts, we enrolled 30 participants in the study.

### Ethical aspects

Ethical approval was obtained from the University of East Anglia Faculty of Medicine and Health Research Ethics Committee in Norwich (UK) and the Ethics Committee of the Nutritional Research Institute (IIN) in Peru. The participants gave written informed consent. The study was registered at www.clinicaltrials.gov (Identifier number NCT05154500).

### Statistical analysis

Calculations and statistical analyses were performed using R version 4.1.2 (R Foundation for Statistical Computing, Vienna, Austria). Data distribution was tested by Shapir–Wilk and were reported as mean ± SD if normally distributed or as median (IQR) if not normally distributed. FIA and TIA values are reported as geometric means (95% confidence interval). Two-sided paired *t*-tests were used for comparisons of iron absorption. Two-sided unpaired *t*-tests were used for the comparisons of test meal composition. *P* values <0.05 and <0.001 were considered significant and highly significant, respectively. In addition, a linear mixed-effects model was used to test for carryover effects (R Core Team (2022)). The R Foundation for Statistical Computing, Vienna, Austria) using the lme4 [22] and ImerTest [23] packages. The outcome variable was the “% of Fe absorbed,” which was analyzed for associations with “meal type” (regular or biofortified) and “sequence” of meal (first or second) and the interaction of "meal type x sequence,” all as fixed effects. The random effect of participant ID was included to account for the crossover nature of the design.

## Results

### Study participants

Thirty participants were recruited for the study. Twenty-nine completed the study according to the protocol. One participant missed endpoint sampling. The data from this participant were, therefore, excluded from the statistical calculations. Baseline characteristics of the participants are shown in Table 1. Hb values were corrected for altitude [12]. Twelve participants were anemic (Hb <12 g/dL) and 17 participants had PF <15 μg/L. Hb, PF, and inflammation status markers did not change in participants over the 6-wk intervention period. At screening in Peru, all participants had a PF concentration <25 μg/L measured by electrochemiluminescence; however, when repeated PF measurements were performed using an automated immunoassay in Zurich, only 12 of 28 participants had PF <25 μg/L. On day 15, the beginning of the second round of meals, 10 out of 28 participants had a PF >25 μg/L as measured by immunoassay. The PF concentrations measured by immunoassay were reported in the manuscript.

**TABLE 1:** Baseline (day 1) characteristics of women who consumed the test meals and were included for final data analysis (*n* = 28). Values expressed as mean ± SD

Parameters	Day 1 (baseline)
Age (y)	23 ± 4.5
Body height (cm)	152.3 ± 4.7
Body weight (kg)	52.3 ± 5.1
Body mass index (kg/m^2^)	22.5 ± 1.8
Hemoglobin (g/dL)^1^	14.4 ± 1.4
Plasma ferritin (μg/L)^2^	21.3 ± 13.3
C-reactive protein (mg/L)	1.1 ± 0.7

1 Hemoglobin values were corrected for altitude [12].

2 Measured using automated immunoassay in Zurich, Switzerland.

### Characterization of potato test meals

The composition of the iron-biofortified and nonbiofortified test meals is summarized in Table 2. The analysis was performed on test meals prepared using a subset of the tubers used for the preparation of the test meals, 2 wk before the preparation of all test meals.

**TABLE 2:** Composition of the test meals. Values-based on fresh weight basis, expressed as mean ± SD unless otherwise indicated

Parameters	Nonbiofortified Peruanita	Biofortified M3-21.75
Fe (mg/100 g)	0.31 ± 0.01	0.63 ± 0.01∗∗
Fe per 460 g meal (mg)	1.43	2.90
Ascorbic acid^1^ (mg/100 g)	9.92 ± 0.74	16.22 ± 0.32∗∗
Ascorbic acid^2^ (mg/100 g)	3.74 ± 0.39	7.65 ± 0.34∗∗
Ascorbic acid^2^: Fe molar ratio	3.82	3.84
Phytic acid (mg/100 g)	3.10 ± 1.72	39.34 ± 3.04∗∗
Phytic acid: Fe molar ratio	0.84	5.28
Total polyphenols (mg/100 g)	32.0 ± 5.8	25.0 ± 3.5
Chlorogenic acid (mg/100 g)	22.51 ± 3.98	15.14 ± 1.72∗
Neochlorogenic acid (mg/100 g)	2.71 ± 0.49	1.06 ± 0.14∗∗
Cryptochlorogenic acid (mg/100 g)	0.31 ± 0.12	0.14 ± 0.04
Caffeic acid (mg/100 g)	0.36 ± 0.10	0.32 ± 0.05
Tryptophan (mg/100 g)	5.88 ± 1.23	8.30 ± 1.59
Rutin (mg/100 g)	0.24 ± 0.02	0.01 ± 0.00∗∗
Isotopic label (mg) per 460 g meal	0.3	0.3
Total weight of consumed potatoes over 10 d (g)	4621 ± 21	4584 ± 33∗∗

∗,∗∗ Significant difference between varieties: ∗ *P* < 0.05, ∗∗ *P* < 0.01

1 In the samples before long storage (5 mo) at −18°C and thawing.

2 In the samples after long storage (5 mo) at −18°C and thawing.

Mean ± SD iron, phytic acid, and ascorbic acid concentrations in the iron-biofortified and the nonbiofortified potato meals (mg/per 100 mg) were 0.63 ± 0.01 and 0.31 ± 0.01, 39.34 ± 3.04 and 3.10 ± 1.72, and 7.65 ± 0.34 and 3.74 ± 0.39, respectively (*P* < 0.01) while chlorogenic acid concentrations were 15.14 ± 1.72 and 22.52 ± 3.98, respectively (*P* < 0.05). Average daily consumption ± SD of the iron-biofortified and the nonbiofortified potato meals was 460 (± 0.5) g, resulting in a mean daily intrinsic iron intake of 2.90 mg from the biofortified and of 1.43 mg from the nonbiofortified potato meals (*P* < 0.01).

### FIA and TIA from the test meal

**FIGURE 3:**
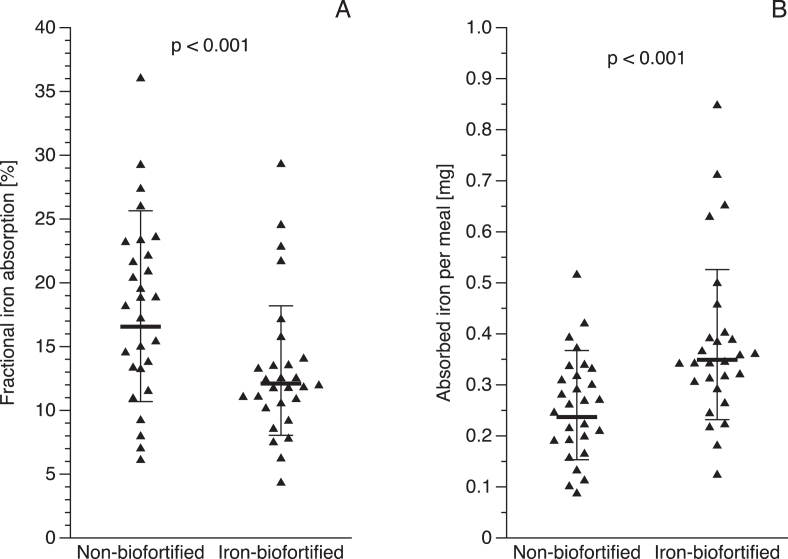
Fractional iron absorbed (%) (A) and total iron absorbed per meal (mg) (B) of nonbiofortified and iron-biofortified potatoes. Isotopic administration: ^57^Fe for the nonbiofortified test meal and ^58^Fe for the iron-biofortified test meal. The horizontal lines represent geometric means ± SD. *n* = 28. Nonbiofortified: yellow-fleshed potato variety Peruanita; biofortified: iron-biofortified yellow-fleshed potato clone.

The FIA (geometric mean; 95% CI) of the biofortified potato clone and the nonbiofortified potato variety were 12.1% (10.3%–14.2%) and 16.6% (14.0%–19.6%), respectively (*P* < 0.001) (Figure 3A), and TIA (geometric mean; 95% CI) from the biofortified clone and the nonbiofortified variety were 0.35 mg (0.30–0.41 mg) and 0.24 mg (0.20–0.28 mg)per 460 g of potato, respectively (*P* < 0.001) (Figure 3B). The FIA of the biofortified clone was on average 37% lower than that of the regular variety (Figure 3A), but because of its higher iron content, 45.8% more iron was absorbed from the biofortified variety than from the regular variety for the same weight of potatoes (Figure 3B). Results from the linear mixed-effects model showed that meal type was highly significantly (*P* < 0.001) associated with %Fe absorbed but the sequence (*P* = 0.8625) and interaction of meal type x sequence (*P* = 0.5375) had no association with %Fe absorbed.

## Discussion

The main finding of this study is that although FIA of the biofortified clone was on average 37% lower than that of the regular variety, because of its higher iron content, 45.8% more total iron was absorbed from the biofortified variety than from the nonbiofortified variety for the same weight of potatoes.

The higher FIA in the nonbiofortified variety could be attributed to its lower phytic acid level compared to the iron-biofortified clone (3.1 and 39.34 mg/100 g, respectively) (*P* < 0.01). Dietary inhibitors such as phytates, polyphenols and calcium, and enhancers such as ascorbic acid and proteins mainly influence iron bioavailability [24]. The PA:Fe molar ratio of the iron-biofortified meal was higher than that in the nonbiofortified meal (5.29 and 0.84, respectively). However, the ascorbic acid:Fe molar ratio for the nonbiofortified potato meal and the iron-biofortified potato meal were similar.

FIA obtained for the variety Peruanita in the present study (16%) is significantly lower than that reported for the same variety in a previous study conducted by our team (28%) [9]. There are 3 possible reasons that could explain the lower FIA in the present study relating to the iron status of participants and differences in levels of dietary inhibitors and enhancers [25].

First, the mean PF in the participants from the present study (20 μg/L) is twice that of the participants in the Jongstra et al. study reported in 2020 (10 μg/L) [9]. Lower iron absorption in the variety Peruanita during 2021 probably reflects the fact that in the present study, participants had higher stores of iron; hence, the efficiency of iron absorption is reduced. Beisegel et al. [26] reported that iron absorption of maize meals was inversely related to the serum ferritin of participants. Similarly, Petry et al. [27] reported a higher FIA for beans in women with low iron status (FIA ranging from 7.1% to 9.2%; mean plasma ferritin of 8.9 μg/L) than in women with higher iron status (FIA ranging from 3.4% to 4.7%; mean plasma ferritin of 14.4 μg/L) [11].

In view of the marked influence of iron status on absorption, previous studies have used normalized FIA values for correcting absorption values to a common reference point [28]. Using the iron status FIA correction equation published by Cook et al. [29], we estimate that the variety Peruanita and the iron-biofortified clone used in this study would have a FIA of around 27% and 20%, respectively, in women with mean serum ferritin 10 μg/L. The 27% FIA estimated for the nonbiofortified variety Peruanita is similar to the value reported in our previous study (28%) [19] for the same variety. Therefore, the difference in iron status of participants in the 2 studies may well be the reason for the difference in FIA values for the same potato variety.

Second, ascorbic acid concentrations in the meals given in the present study were lower than in meals given by Jongstra et al. [9], probably because of differences in the preparation of potato meals between the studies. In Jongstra et al.’s study, potato meals (boiled, peeled, and mashed tubers) were prepared and consumed on the same day, whereas in the present study conducted in 2021, because of Covid-19 restrictions, the feeding trials had to be postponed by 4 mo (from May to August) and the meals were prepared and stored frozen at −20°C until they were consumed. Before consumption, the frozen samples were allowed to thaw at room temperature overnight. The process of freezing and thawing was associated with a reduction of ascorbic acid from 9.92 to 3.74 mg/100 g FW in the nonbiofortified potato variety Peruanita. The presence of ascorbic acid in the diet increases the absorption of nonnonheme iron [30]. Ascorbic acid can reduce ferric iron to ferrous iron, which is the only form of iron that can be absorbed via iron transporters of intestinal enterocytes, and it also can chelate ferric iron, making it soluble at the slightly alkaline pH of the duodenum [31]. Hence, it is possible that lower iron absorption may have been partially caused by the reduction in ascorbic acid.

Third, the level of the most abundant polyphenol (chlorogenic acid) in Peruanita tubers in our study (22.51 mg/100 g, fresh weight) was significantly higher compared with the levels in Peruanita tubers used by Jongstra et al. [9] (8.59 mg/100 g, FW). Dietary polyphenols, including phenolic acids such as chlorogenic acid that contains a dihydroxy catechol group, are inhibitors of iron absorption [32], hence a higher level of chlorogenic acid could contribute toward the lower observed iron bioavailability from the tubers.

The FIA of both the commercial variety Peruanita and the biofortified potato clone was above the expected 5% to 10% for plant-based diets [33], in which phytic acid is the main inhibitor of nonheme iron absorption [34]. The potato meals provided in this study (which represent the typical consumption in rural areas of Peruvian highlands) contain lower phytic acid levels (15.5–196 mg/meal) than the bean meals (529–702 mg/meal) provided by Petry et al. [27] in a study examining iron absorption from beans, and the pearl millet meals (392–511 mg/meal) provided by Cercamondi et al. [10] in a study measuring iron absorption from pearl millet. The PA:Fe molar ratio in the potato meals is also lower (<6:1) than that reported for bean meals (>10:1) [27], suggesting higher iron bioavailability of potatoes compared with beans.

TIA from the iron-biofortified potato clone was higher than the commercial variety (0.38 vs 0.26 mg per 500 g potato meal, respectively). TIA from the iron-biofortified potato was similar to TIA from the iron-biofortified bean meal (0.41 mg) evaluated in Petry et al. [25] but lower than TIA from the biofortified pearl millet meal (1.13 mg) evaluated in Cercamondi et al. [10]. However, is important to note that in the present study, the mean plasma ferritin of the women (21.3 μg/L) was not as low as for the bean and pearl millet study (8.9 μg/L and 11.9 μg/L, respectively). In consequence, it is expected that FIA and TIA from iron-biofortified potatoes will be higher in participants with lower levels of iron stores.

In the rural areas of the highlands of Peru and Latin America, women consume meals containing only potatoes and have a mean potato consumption >500 g per day [6]. Consumption of 500 g of iron-biofortified potato provides 27% of the iron requirement (1.41 mg per day, according to IOM, 2001 [35] for absorbed iron) in women living in these areas with moderate iron stores (determined by plasma ferritin levels around 20 μg/L) and could provide >50% of the requirement for women with low iron stores (PF around 10 μg/L). However, in areas where potato consumption is lower, the contribution of iron in potatoes to the iron requirement would be lower. For example, in the urban areas of the Peruvian highlands, the mean potato consumption is around 200g. In this case, potatoes would provide around 10% of the iron requirement of women with moderate iron stores and around 20% of women with low iron stores.

The strength of this study is that we used well-established stable isotope techniques and gave multiple meals, which reduces day-to-day variability in iron absorption, to accurately measure iron absorption and assess iron bioavailability from the 2 varieties of potato. The results obtained in this study are a prerequisite for further research on biofortified potatoes, in particular efficacy trials investigating changes in iron status with long-term intake of iron-biofortified potatoes. A weakness is that the findings are restricted to communities where the potato is a major component of the diet, namely, in rural areas of Latin-American highlands. Iron absorption in meals that combine potatoes with other foods such as beans, as in African rural areas, might produce different findings depending on the concentration of iron promoters and inhibitors in the diet.

In conclusion, FIA from both the iron-biofortified and nonbiofortified potato meals were above the expected values of 5% to 10% for plant-based diets. The absorbed iron from the iron-biofortified potato meal was higher than that from the nonbiofortified potato meal. In areas of the Peruvian highlands where potato consumption is high (500 g), iron-biofortified potatoes would provide 27% of the daily requirement for absorbed iron in women with moderate iron stores and above 50% in women with low stores. Iron-biofortified yellow-fleshed potatoes provide a significant amount of absorbable iron and can contribute to reduce iron deficiency in rural areas of the Latin-American highlands where potato consumption is high and iron deficiency is prevalent.

## Funding

This research was funded by the Biotechnology and Biological Sciences Research Council (BBSRC) Global Challenges Research Fund (GCRF) Grant No. BB/S014039/1. We gratefully acknowledge USAID Feed the Future Crops to End Hunger award to CIP (DIS-B-AID-BFS-IO-17-00005).

## Author disclosures

The authors report no conflicts of interest.

## Author contributions

The authors’ responsibilities were as follows – GB, RL, SFT, MZ, CZ, TZF, MBZ, RM: conceived the study; GB, RL, SFT, MP, CZ, RM, PAK, RFH, JD: developed the research protocol; GB, RL, SFT, MP, CZ, PAK, OAJ, GH, EB: provided guidance; GB, RL, CZ: conducted the research and analyzed the data; ES, TZF: organized the production of the iron-biofortified and nonbiofortified potatoes; GB: wrote the manuscript; GB, SFT: had primary responsibility for its final content; all authors contributed to the design of the study and read and approved the final manuscript.

## Acknowledgments

The authors thank Universidad Nacional de Huancavelica and Instituto Pedagogico de Huancavelica for their support in recruiting the participants. We thank all participating women and field workers for their contribution, in especial to Sofia Huanuco, Maria Fernanda Puch and Nadine Paz Soldan for their fieldwork supervision, and Paola Sosa for organizing the potato meal preparation.Data described in the manuscript will be made available upon request pending application and approval.

## Data Availability

Data described in the manuscript will be made available upon request pending application and approval.
